# The Higher Sensitivity of GABAergic Compared to Glutamatergic Neurons to Growth-Promoting C3bot Treatment Is Mediated by Vimentin

**DOI:** 10.3389/fncel.2020.596072

**Published:** 2020-11-03

**Authors:** Andrej Adolf, Paul Turko, Astrid Rohrbeck, Ingo Just, Imre Vida, Gudrun Ahnert-Hilger, Markus Höltje

**Affiliations:** ^1^Institute of Integrative Neuroanatomy, Charité—Universitätsmedizin Berlin, corporate member of Freie Universität Berlin, Humboldt-Universität zu Berlin, and Berlin Institute of Health, Berlin, Germany; ^2^Institute of Toxicology, Hannover Medical School (MHH), Hannover, Germany

**Keywords:** C3 transferase, vimentin, GABAergic neurons, glutamatergic neurons, axon outgrowth

## Abstract

The current study investigates the neurotrophic effects of *Clostridium botulinum* C3 transferase (C3bot) on highly purified, glia-free, GABAergic, and glutamatergic neurons. Incubation with nanomolar concentrations of C3bot promotes dendrite formation as well as dendritic and axonal outgrowth in rat GABAergic neurons. A comparison of C3bot effects on sorted mouse GABAergic and glutamatergic neurons obtained from newly established *NexCre*;*Ai9xVGAT Venus* mice revealed a higher sensitivity of GABAergic cells to axonotrophic and dendritic effects of C3bot in terms of process length and branch formation. Protein biochemical analysis of known C3bot binding partners revealed comparable amounts of β1 integrin in both cell types but a higher expression of vimentin in GABAergic neurons. Accordingly, binding of C3bot to GABAergic neurons was stronger than binding to glutamatergic neurons. A combinatory treatment of glutamatergic neurons with C3bot and vimentin raised the amount of bound C3bot to levels comparable to the ones in GABAergic neurons, thereby confirming the specificity of effects. Overall, different surface vimentin levels between GABAergic and glutamatergic neurons exist that mediate neurotrophic C3bot effects.

## Introduction

Monomeric GTPases of the Rho family are important cellular switches that regulate neuronal morphology. RhoA usually functions as a negative regulator of axon outgrowth and together with RhoB and C can be inhibited by *Clostridium botulinum* C3 exoenzyme (C3bot) that represents the prototype of bacterial ADP-ribosyltransferases (Aktories and Frevert, 1987). For this reason, C3bot and derivatives have been extensively used as tools to investigate neurite outgrowth and to foster neuronal plasticity for the potential treatment of CNS traumas like spinal cord injuries (SCI). This has been investigated in different animal models (Dubreuil et al., 2003; Boato et al., 2010; Loske et al., 2012). Moreover, systematic reviews and meta-analyses (Watzlawick et al., 2014), as well as clinical trials in humans, have subsequently confirmed these findings (McKerracher and Anderson, 2013; Fehlings et al., 2018).

Despite the lack of a classical cell-binding subunit of C3bot (as in the case of the clostridial neurotoxins), we were able to demonstrate that the intermediate filament protein vimentin as well as the ß1-subunit of integrins, known to serve as a transmembrane receptor for cell-extracellular matrix adhesion, function as surface interaction partners for C3bot (Rohrbeck et al., 2014, 2017; Adolf et al., 2019). Lack of vimentin results in failure of C3bot to foster axonal outgrowth in hippocampal/neocortical neurons as shown in vim^−/−^ cultures (Adolf et al., 2016). In wild type neurons surface vimentin might originate from astrocytic exosomal release followed by binding to neuronal membranes but might also stem from so far unraveled neuronal trafficking routes guiding this primarily intracellular protein to the cell surface. Astroglial upregulation and subsequent vimentin release *in vivo* has also been demonstrated by other groups in the intact and injured CNS (Teshigawara et al., 2013; Shigyo and Tohda, 2016). In this context, we were able to show that exosomes obtained from cultured scratch-injured wild type but not vim^−/−^ astrocytes enhanced binding of C3bot to synaptosomes from the brain and spinal cord (Adolf et al., 2019). Generally, studies to investigate neuronal process outgrowth and regeneration by C3bot primarily used mixed neuronal cultures containing different neuronal subtypes as well as co-cultured glia cells. Consequently, the observed promoting effects of C3bot on axonal and dendritic growth reflect the net outcome of a plethora of neuron subtypes in the presence of glia. Also, the regeneration-promoting CNS effects of C3bot or C3bot-derived peptides on axon regeneration *in vivo* in defined neuron types such as the primarily glutamatergic retinal ganglion cell axons in the optic nerve (Bertrand et al., 2007; Hu et al., 2007) as well as glutamatergic corticospinal or serotonergic descending brainstem fibers (Boato et al., 2010) display the effects in a glial environment.

A recently developed culturing method established to obtain highly purified GABAergic neurons from transgenic rat and mice (Turko et al., 2019) together with cultures of highly purified glutamatergic neurons (Goebbels et al., 2006) now opens the opportunity to independently analyze the sensitivity of these two major neuron subtypes to the direct growth-promoting effects of C3bot in the absence of glia. By crossing glutamatergic TdTomato expressing *NexCre*;*Ai9* and GABAergic *VGAT-Venus mice* a transgenic *NexCre*;*Ai9xVGAT-Venus* mouse line was obtained that offers the advantage to obtain highly purified FAC sorted neuronal cultures of both neuron subtypes from littermates or even the same animal.

The current study was undertaken, first, to determine if putative differences in expression levels of neuronal C3bot binding partners such as ß1-integrin and vimentin exist that could mediate differential responsiveness to C3bot in inhibitory and excitatory neuron types. Second, we wanted to answer the question if neuronal vimentin, in the absence of a glial source, is sufficient to mediate C3bot effects. Therefore, we compared the effects of C3bot on axonal and dendritic growth of GABAergic and glutamatergic neurons by morphometrical analysis. Biochemically, we determined the expression levels of vimentin and ß1-integrin as well as the ability of C3bot to bind to these two cell types.

## Materials and Methods

### Expression of Recombinant C3bot and Vimentin Protein

*Clostridium botulinum* C3 protein (C3bot) was expressed as recombinant glutathione S-transferase-fusion proteins in E. coli TG1 harboring the respective DNA fragment in the plasmid pGEX-2T. GST was removed by thrombin cleavage. Vimentin expression and purification were performed as described before (Rohrbeck et al., 2014). Plasmids of full-length mouse vimentin provided by Prof. Dr. Yi-Ling Li, Institute of Biomedical Sciences, Genomics Research Center, Academia Sinica, Taipei, Taiwan were used.

### Animals

VGAT-Venus-A Wistar rats that selectively express a yellow fluorescent protein variant (Venus) in more than 95% of cortical GABAergic neurons were used to investigate the effects of C3bot on rat GABAergic neurons (Uematsu et al., 2008). NexCre;Ai9 × VGAT Venus mice were bred from NexCre;Ai9 mice (Goebbels et al., 2006; Madisen et al., 2010; described in Turko et al., 2019) and VGAT Venus mice (Wang et al., 2009). NexCre;Ai9 × VGAT Venus mice express the red fluorescent protein, TdTomato, in the majority of post-mitotic glutamatergic neurons and the yellow fluorescent protein, Venus, in the majority of GABAergic and glycinergic neurons. Genotyping of pups before cell culture (Figure 2A) was done using a fluorescent head-mounted miner’s lamp (Excitation filter FS/ULS-02B2/emission filter FS/TEF-3GY1: BLS Ltd, Budapest, Hungary).

**Figure 1:**
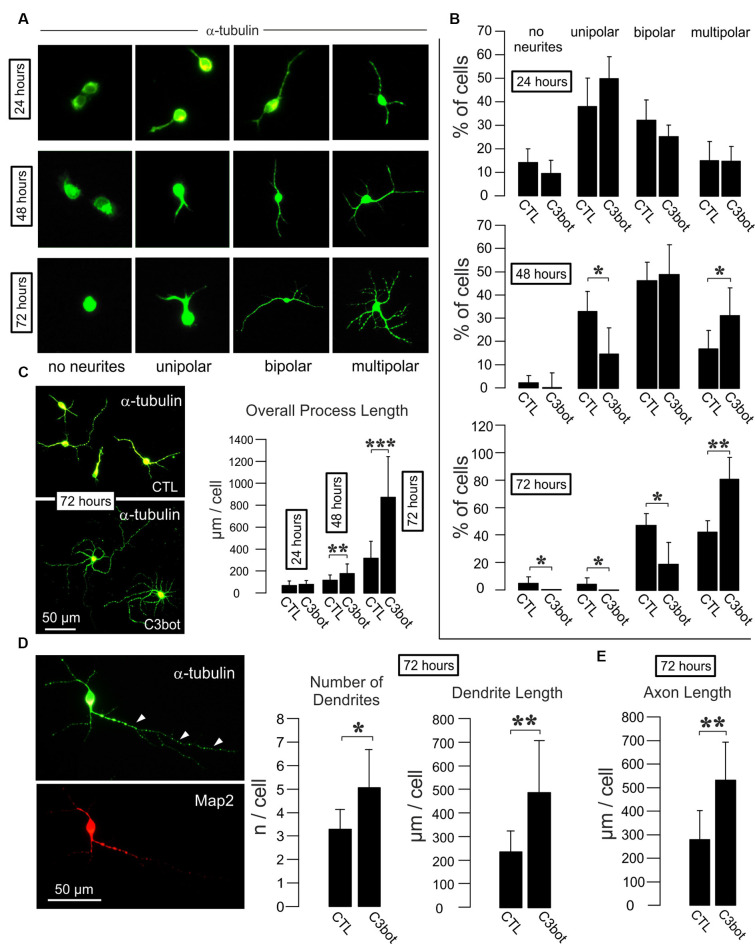
C3bot fosters dendrite formation and outgrowth and enhances axon length of purified rat GABAergic neurons. **(A)** Representative images of GABAergic neurons stained for α-tubulin displaying the respective morphologies at the indicated time points. Images not to scale. **(B)** Positive-sorted GABAergic neurons obtained from VGAT-Venus rats were incubated at low density for 24, 48, or 72 h (DIV1–DIV4) with or without 300 nM of C3bot application. Bar charts summarizing the effect of C3bot on GABAergic cell morphology over time. After 48 h, C3bot-treated neurons showed a significantly enhanced proportion of multipolar cells paralleled by a reduced number of unipolar cells. One day later, C3bot-treated cells had increased the multipolar phenotype 2-fold compared to control neurons, reflected by a decreased proportion of bipolar, unipolar, and cells bearing no processes. **(C)** Fixed cells were subjected to morphometrical analysis of overall process length after 24, 48, or 72 h of incubation with 300 nM of C3bot. Significant promoting effects were observed after 48 h and, more pronounced, after 72 h of treatment. **(D)** Fixed cells were subjected to morphometrical analysis of dendrite number and overall dendrite length after 72 h of C3bot incubation. Map2 staining was used to support the differentiation of neuronal processes into dendritic and axonal (arrowheads) compartments. Both parameters were significantly increased by C3bot compared to untreated cells. **(E)** Axon growth was also analyzed and revealed the promoting effect of C3bot on axon length. Data represent the means ±SEM. Statistical differences were determined by Student’s *t*-test (*p ≤ 0.05; **p ≤ 0.01; ***p ≤ 0.001). For 24, 48, and 72 h incubation time, data were obtained from a total of 251, 331, or 161 neurons, respectively. Per condition, cells from three individual coverslips and two animals were evaluated.

**Figure 2:**
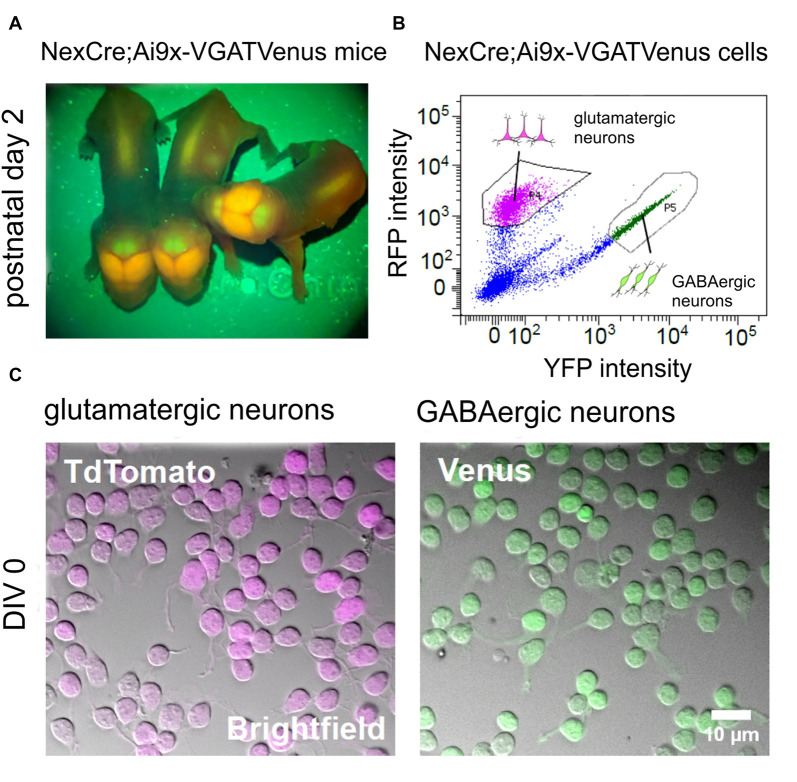
Generation of purified GABAergic and glutamatergic cell cultures using fluorescence-based cell sorting. **(A)** Images showing the fluorescent signal from transgenic *NexCre;Ai9xVGAT Venus* mice at postnatal day 2. The Neocortex appears strongly positive for TdTomato, due to the large numbers of glutamatergic neurons in this area. The cerebellum appears strongly positive for Venus, due to the large numbers of Purkinje and other GABAergic neurons in the molecular layer. **(B)** Intensity scatter plot of *NexCre;Ai9xVGAT Venus* dissociated cortico-hippocampal cells. Strongly fluorescent TdTomato (glutamatergic) or Venus (GABAergic) cells were selected for sorting (see gating boxes). **(C)** Combined infrared (bright field) images and superimposed fluorescent signals from living TdTomato (left) and Venus (right) positive neurons after 1 h *in vitro*.

### Cell Sorting of GABAergic and Glutamatergic Neurons

The purification and culture of GABAergic and glutamatergic neurons have been described previously (including a Jove video protocol of the whole procedure, found in Turko et al., 2019). In brief, cortico-hippocampal tissue from postnatal (days 0–2) VGAT-Venus-A Wistar rats or NexCre;Ai9 × VGAT Venus mice (Figure 2A) was dissected and dissociated in papain (1.5 mg/ml for 30 min). Cells were then resuspended in Hibernate A low fluorescence transport medium (BrainBits, Springfield, IL, USA), supplemented with B27 (1× concentration), Glutamax (1× concentration), and penicillin-streptomycin (100 U/ml; all sourced from Gibco, Waltham, MA, USA). Dissociated cells were then sorted using either BD FACSAria I or II flow cytometers, at the Flow Cytometry and Cell Sorting Facility (FCCF) in the Deutsches Rheuma-Forschungszentrum (DRFZ, Berlin Germany). An example dot plot showing the typical gating parameters used for sorting is given in Figure 2B. Following sorting, purified GABAergic and glutamatergic neurons were resuspended in Neural Basal A (NBA) medium (with the same supplements as Hibernate A medium; Thermo Fisher Scientific, Waltham, MA, USA). For cell morphology analysis and immunocytochemistry, purified neurons were plated onto 12 mm, round, glass coverslips in a 24-well plate, at a density of 1,000 cells/μl, 10 μl/well. For Western blot experiments, cells were plated in six-well plates, at a density of 500 cells/μl, 2 ml/well (1 × 10^6^ cells per well). Coverslips and culture plates were coated in poly-L-lysine hydrobromide solution before plating (20 μg/ml, 1 h; Merck Millipore, Darmstadt, Germany). Cells were grown for 4–14 days *in vitro* (DIV) before direct fixation in 4% paraformaldehyde (PFA) solution for cell morphology and immunocytochemistry analysis or harvested without fixation for protein analysis. Cells were incubated at 37°C, at 5% CO_2_, in a humidified incubator (Heraeus; Thermo Fisher Scientific, Waltham, MA, USA).

### Antibodies

#### Primary Antibodies

Monoclonal mouse anti-Tau IgG (#314011, IF dilution 1:1,000, Adolf et al., 2016), polyclonal rabbit anti-microtubule-associated protein 2 (Map2, #188003, IF dilution 1:1,000, Troca-Marín et al., 2011), polyclonal rabbit anti-vimentin (#172002, IF dilution 1:400, Adolf et al., 2019) and polyclonal rabbit ß1-integrin IgG (#240003, WB dilution 1:1,000; Liu et al., 2009) were from Synaptic Systems, Göttingen, Germany. A monoclonal rat anti-ß1-integrin IgG (#MAB 2405, IF dilution 1:500) was from R&D Systems (Minneapolis, MN, USA). An affinity-purified polyclonal rabbit IgG against full-length C3bot (IF dilution 1:1,000, WB dilution 1:2,000) was developed in our lab (Rohrbeck et al., 2014). A monoclonal mouse anti-glyceraldehyde-3-phosphate dehydrogenase IgG (GAPDH, MAB374, WB dilution 1:4,000, Dazard et al., 2003) was from Merck Millipore.

#### Secondary Antibodies

Alexa 594 goat anti-rabbit IgG (#11012, dilution 1:500), Oregon Green goat anti-rabbit IgG (#0-11038, dilution 1:300), Oregon Green goat anti-mouse IgG (#0-6380, dilution 1:500) were from Invitrogen (Carlsbad, CA, USA). Alexa 647 goat anti-rabbit IgG (#111-605-144) was from Jackson Labs, Bar Harbor, USA. Peroxidase-conjugated horse anti-mouse IgG (#PI-2000. Dilution 1:4,000) and goat anti-rabbit IgG (#PI-1000, dilution 1:1,000–4,000) were from Vector Laboratories, Burlingham, NY, USA.

### Immunofluorescence

Cultured neurons were fixed after 4 days in culture with 4% formalin in 0.1 M PBS and incubated at 4°C overnight with the respective primary antibodies (see above). Following repeated washing in PBS, cells were incubated for 2 h at RT with fluorescence-conjugated secondary antibodies. Cells were again washed and mounted with Immu-Mount^®^ (Thermo Fisher Scientific, Waltham, MA, USA).

For lectin-based membrane staining, an Alexa Fluor 488-conjugated wheat germ agglutinin (Thermo Fischer Scientific, Waltham, MA, USA) was used. Typically, the conjugate was added by a medium application at 2.5–5 μg/ml for 10 min at 36°C. Cells were washed 3× with PBS before further handling. Images were acquired using epifluorescence (see below) or using a Leica SL confocal device.

### Morphometrical Analysis

**Figure 3:**
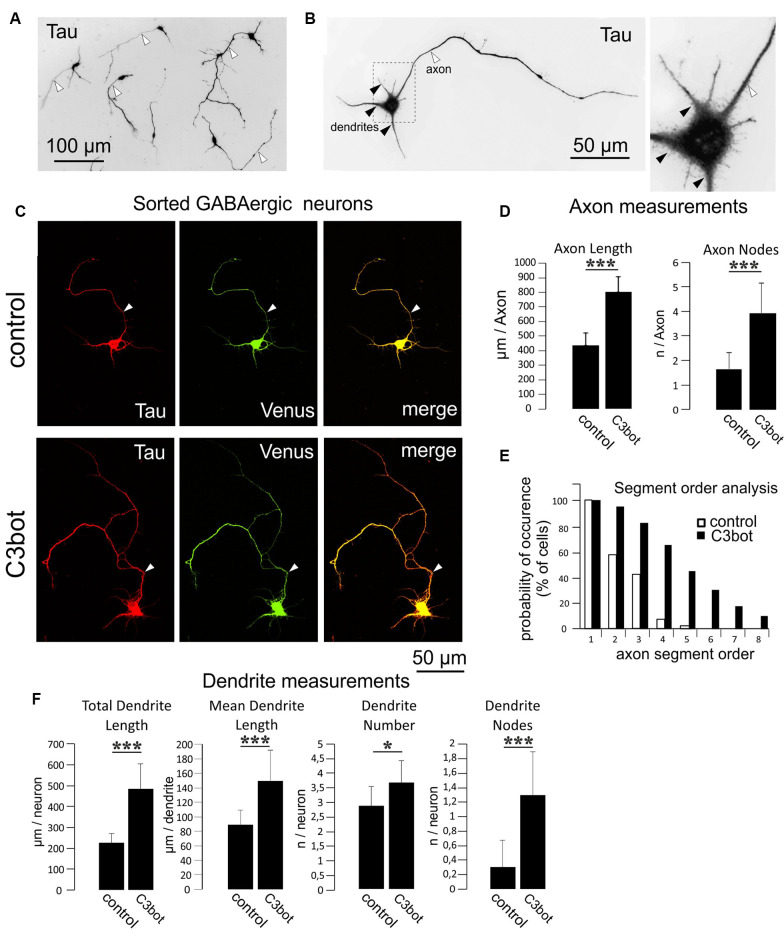
C3bot-mediated neurotrophic effects on purified GABAergic neurons. **(A)** Positive-sorted GABAergic neurons obtained from *NexCre;Ai9x VGAT Venus mice* were grown at low density for 72 h (DIV1–DIV4), fixed and immunostained for Tau indicating neuronal morphology. Most neurons had developed processes differentiated into a single axon (white arrowhead) and several dendrites. **(B)** GABAergic neurons at higher magnification. A single axon (white arrowhead) and several shorter tapering dendrites (black arrowheads) extend from the soma. **(C)** Positive-sorted GABAergic neurons were grown at low density for 72 h (DIV1–DIV4) with or without 300 nM of C3bot application. Following incubation, cells were fixed and immunostained for Tau strongly labeling axons and dendrites. A GABAergic cell phenotype was ascertained from endogenous Venus expression. **(**Arrowhead: axon, **D)** Fixed cells were subjected to morphometrical analysis of axon length and branch nodes. Both parameters were significantly increased by C3bot treatment relative to controls. **(E)** Axon segment order analysis revealed that treatment with C3bot increased branching up from second-order branches. **(F)** Morphometrical analysis of dendrite parameters. Treatment with C3bot resulted in increased dendritic length, number, and branch nodes. Data represent the means ± SEM. Statistical differences were determined by Student’s *t*-test (*p ≤ 0.05; ***p ≤ 0.001). Per condition, a representative analysis of a total of 40 neurons from four individual coverslips and two animals is displayed.

Total length and the overall number of branches from axons and dendrites, as well as axon segment orders (first order represents the axon stem, second order the branches originating from the first branch node and so on), were analyzed morphometrically from images taken by a Leica DMLB upright epifluorescence microscope using a PL FLUOTAR 40× objective using the Neurolucida software applying manual reconstruction (MicroBrightField, Williston, VT, USA). Filter cubes were the following: 450–490 nm excitation with a 510 nm dichromatic mirror, 515–560 nM excitation and 580 nm dichromatic mirror and 620–660 nm excitation and 660 nm dichromatic mirror for green, red, and invisible red spectrum fluorescence, respectively. The parameter “axon length” represents the integral length of all visible parts of an axon, including branches. Typically, 30–40 neurons per condition were randomly chosen given they exhibited a clear morphological differentiation into the axonal and dendritic compartment as judged mainly by the length of the neurites (see also Figures 3A,B) and evaluated from independent experiments that were carried out two to three times. Exact *n* values for cells, coverslips, and animal numbers are given in the respective figure legends. For morphometrical reconstruction Tau staining was applied that exclusively marks axons in mature neurons but also works as an excellent antibody to stain neurites up to the fine branches of both the axonal and the dendritic compartment in immature neurons used in our study. Providing there were no significant differences between coverslip means (means of the axon and dendrite parameters from individual coverslips), data were pooled and given as mean ± SEM. Unless otherwise stated, a Student’s *t*-test was used to test for significance between means [significance: 0.05 (*), 0.01 (**), and 0.001 (***)].

### C3 Binding Assays

To biochemically assess C3 binding, cultured neurons were first grown for 7 days *in vitro* (DIV). Typically, 2–5 × 10^6^ neurons were used per condition, in each experiment. To test for C3bot interaction with specific neuron types, cell culture supernatant was first discarded and cells were harvested in PBS. Suspended cells were exposed to 300 nM of C3bot for 1 h at 4°C with or without addition of 1 ng/μl recombinant vimentin. Subsequently, cells were washed two times with PBS. Cells were then pelleted and transferred into the Laemmli sample buffer for gel electrophoresis and blotting. For immunocytochemical analysis of C3bot binding, purified GABAergic and glutamatergic neurons were cultured together in a 50:50 mix (500 cells YFP and 500 cells RFP/μl in a 10 μl droplet—total cells = 10 × 10^4^ cells/coverslip). Cells were grown to 7 DIV before 1-h treatment with 300 nM C3bot (4°C). Cells were then washed twice in PBS before fixation and processing for immunocytochemistry.

## Results

To investigate whether C3bot affects GABAergic interneurons in the absence of glial cells, we first analyzed GABAergic neuronal cultures obtained from *VGAT-Venus-A* Wistar rats FAC sorted cultures of these animals selected for Venus-positive cells contain 99% of GABAergic neurons (Turko et al., 2019). FAC sorted cultures of these animals selected for Venus-positive cells contain 99% of GABAergic neurons (Turko et al., 2019). For a readout, we looked at the morphological effects elicited by medium application of *Clostridium botulinum* C3 transferase (C3bot) on neocortical/hippocampal neurons prepared from early postnatal brains. Initially, we started with analyzing the C3bot-mediated effects on neurite formation and early growth during the first 72 h (Figures 1A,B). After 24 h in culture, the majority of cells presented as unipolar neurons with no significant effects of C3bot observable. A day later C3bot had significantly lowered the number of unipolar neurons but raised the proportion of multipolar cells. After 72 h the majority of control cells exhibited either a bipolar (50%) or multipolar (40%) morphology. Treatment with C3bot, on the other hand, promoted the formation of multipolar cells that represented the vast majority of neurons (80%) while the number of bipolar cells was reduced to 20%. Next, we analyzed the overall length of the neuronal processes (Figure 1C). Starting from 48 h of incubation, C3bot treatment resulted in a significant promotion of process growth reaching nearly +175% after 72 h. To see whether the effect of an increased process formation resulted from enhanced dendrite formation in C3bot-treated neurons we stained the neurons for the dendritic marker microtubule-associated protein 2 (Map2) and counted the number of dendritic processes. The morphometrical analysis indeed revealed a significantly increased number of dendrites in treated neurons (Figure 1D). Differential analysis of dendritic vs. axonal effects (only neurons showing a clear morphological differentiation in a dendritic and axonal compartment, see also Figure 3A for general criteria applied, were analyzed) revealed that both dendritic and axonal growth contributed to the observed effect (Figures 1D,E).

Taken together, the results demonstrate a direct and glia-independent growth-promoting effect of C3bot on a defined subpopulation of neurons, namely neocortical/hippocampal GABAergic interneurons.

We then asked whether different subtypes of neurons exhibit differential sensitivity to the inhibition of rho-mediated pathways by C3bot. To this end we used newly established transgenic *NexCre;Ai9xVGAT* Venus mice that offer the opportunity to obtain purified GABAergic and glutamatergic neurons from a single animal line or even individual animals. Consequently, this allows for a direct comparison of C3bot effects on these neuronal subtypes. Animals of glutamatergic *NexCre;Ai9-x* and GABAergic *VGAT-Venus* lines were crossed. *NexCre;Ai9xVGAT-Venus* positive mice were identified by transcranial detection of red and green fluorescence expression after birth using a fluorescent lamp (Figure 2A). A large number of glutamatergic neurons such as the pyramidal cells in the neocortex together with the GABAergic interneurons exhibited a red/orange fluorescence, while the cerebellum appeared green due to a large number of GABAergic neurons in the Purkinje cell layer and Golgi cells in the molecular layer. Following cell sorting (Figure 2B) Venus and TdTomato expressing neurons were enriched to high purity and cultured in isolation (Figure 2C).

Separate cultures of fluorescent Venus (GABAergic) or TdTomato (glutamatergic) neurons obtained by this procedure were used for the morphometrical measurements of C3bot effects on specific neuronal subtypes. To quantify the morphological effects elicited by C3bot we first used GABAergic neurons incubated for 72 h with 300 nM of C3bot. For visualization, endogenous Venus fluorescence was combined with staining for Tau to identify both the axon and dendrites to the finest branches. Tau represents an established axonal marker in cultured mature neurons (Kosik and Finch, 1987) but in the case of the immature neurons used in our study Tau has not yet fully disappeared from dendrites. After 4 days in culture, the vast majority of purified GABAergic neurons had developed a single long process that by characteristic morphological parameters- primarily the length- but also others such as more extensive branching and initial small diameter was easily discernable from dendrites (Figures 3A,B). Morphometrical measurements revealed a C3bot-mediated increased axonal length by 83% (Figures 3C,D). Axonal branching was even more enhanced up from second segment order branches with a total increase of axon nodes by 138% (Figures 3D,E). Analysis of dendrite growth and branching (Figure 3F) revealed C3bot-mediated promoting effects on total dendrite length by 115%, the individual dendrite length was raised by 69%, the number of dendrites by 27%, and the at this maturation stage still poorly developed dendritic branch nodes by 333%. These data supported our previous findings in the rat model.

**Figure 4:**
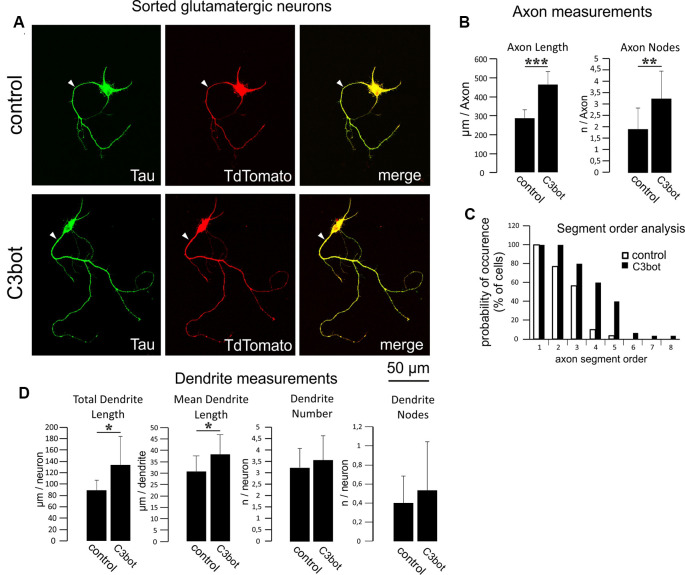
C3bot-mediated neurotrophic effects on purified glutamatergic neurons. **(A)** Positive-sorted glutamatergic neurons were grown at low density for 72 h (DIV1–DIV4) with or without 300 nM C3bot application. Following incubation, cells were fixed and immunostained for Tau as an axonal marker. A glutamatergic cell phenotype was ascertained by endogenous TdTomato expression. **(**Arrowhead: axon, **B)** Fixed cells were subjected to morphometrical analysis of axon length and branch nodes. Both parameters were significantly increased by C3bot treatment relative to controls. **(C)** Axon segment order analysis revealed that treatment with C3bot increased branching up from second-order branches. **(D)** Morphometrical analysis of dendrite parameters. Treatment with C3bot resulted in increased dendritic length. Data represent the means ± SEM. Statistical differences were determined by Student’s *t*-test (*p ≤ 0.05). Per condition, a representative analysis of a total of 30 neurons from three individual coverslips and two animals is displayed. **p ≤ 0.01; ***p ≤ 0.001.

We then applied the same criteria for a morphometrical analysis using purified TdTomato expressing glutamatergic neurons (Figure 4A). The axonal length was increased by 62% following C3bot treatment (Figure 4B). Axonal branching was also enhanced up from second segment order branches with a total increase of axon nodes by 70% (Figures 4B,C). Analysis of dendrite growth and branching revealed significant yet lower C3bot-mediated effects on total dendrite length of 49%, and individual dendrite length of 24%. In contrast to GABAergic neurons, the number of dendrites as well as their branching were not altered (Figure 4D).

In summary, regarding most parameters investigated GABAergic neurons exhibited a higher sensitivity to the neurotrophic effects elicited by C3bot than glutamatergic ones (for a summary, see Table 1).

**Table 1:** Summary of C3bot effects on the morphology of GABAergic and glutamatergic neurons.

	Axon length	Axon nodes	Dendrite length	Mean dendrite length	Dendrite nodes	Number of dendrites
Sorted GABAergic neurons	183%(*)	237, 6%(*)	215%(*)	168, 9%(*)	433%	127, 2%
Sorted glutamatergic neurons	161, 7%	170%	149, 4%	124, 3%	133, 32% (n.s.)	107, 23% (n.s.)

Values are given in % of untreated control cells. Asterisks behind effects on GABAergic neurons indicate statistical significance over effects on glutamatergic neurons (*p ≤ 0.05). n.s.: not significant effects within glutamatergic neurons.

**Figure 5:**
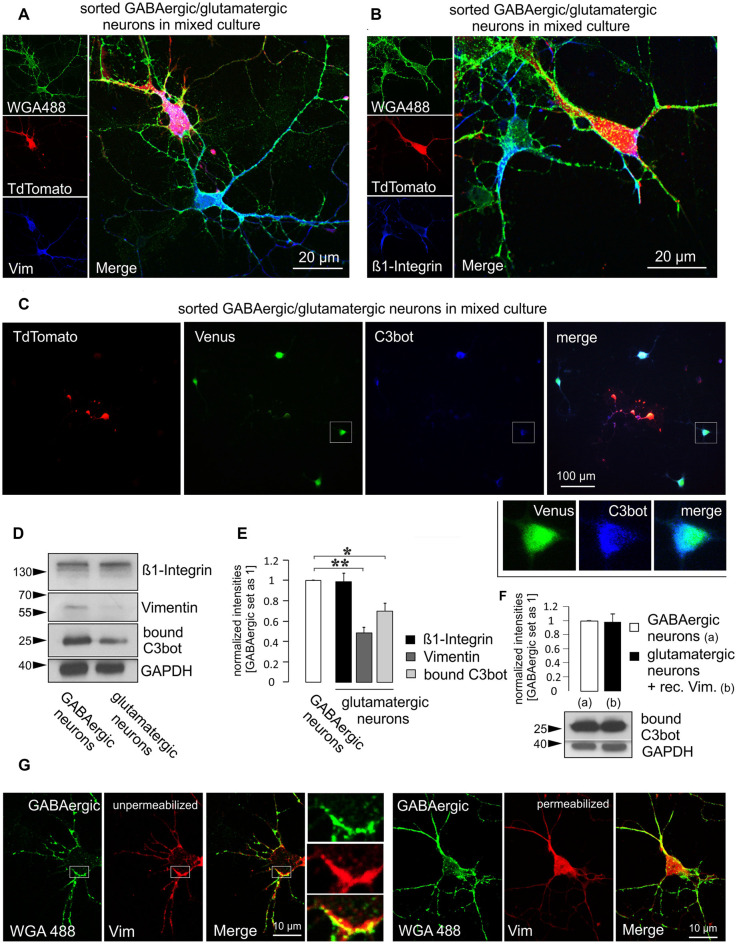
Higher vimentin levels in purified GABAergic neurons are accompanied by increased binding of C3bot. Purified GABAergic and glutamatergic neurons were mixed at a 1:1 ratio and cultured together for 5 days, fixed and immunostained for **(A)** vimentin or **(B)** ß1-integrin using an Alexa647-conjugated secondary antibody. Prior staining of living cells with Alexa-488 conjugated wheat germ agglutinin (WGA488) was used to mark neuronal membranes. TdTomato expression was used to discern glutamatergic from GABAergic cells. Both vimentin and ß1-integrin were expressed at detectable levels in either cell type in the soma and throughout the processes. **(C)** Purified mixed neurons were cultured together for 5 days. Cells were then incubated with 300 nM of C3bot for 1 h at 4°C. Following washing and fixation cultures were stained for bound C3bot. A comparison between Venus expressing GABAergic and TdTomato expressing glutamatergic neurons revealed a stronger association of the C3bot signal (detected by an Alexa 647-coupled secondary antibody) with GABAergic neurons. **(D)** Separate GABAergic and glutamatergic neuron cultures were incubated with 300 nM of C3bot for 1 h at 4°C. Western blot analysis was performed to detect endogenous ß1-integrin and vimentin as well as bound C3bot. GAPDH was used as a loading control. **(E)** A quantitative analysis of the blots shown in **(D)** revealed equal amounts of ß1-integrin, but strongly reduced vimentin levels in glutamatergic neurons compared to GABAergic cells. Accordingly, C3bot binding also was reduced in glutamatergic cells. Data represent the mean of three independent experiments ± SEM. Statistical differences were determined by Student’s *t*-test (*p ≤ 0.05; **p ≤ 0.005). **(F)** Binding assays were performed as in **(D)**, not **(C)** using a modified combinatory treatment with 300 nM C3bot and recombinant vimentin at 1 ng/μl in glutamatergic neurons. Differences in C3bot binding were no longer observable. Data represent the mean of three independent experiments ±SEM. **(C–E)** Data were obtained from three individual experiments. **(G)** Depicted are GABAergic neurons at DIV 5 in mixed culture stained live with WGA488 to mark the plasma membrane. Cells were then fixed and stained for vimentin (Alexa 647-coupled secondary antibody) under permeabilizing (standard) or non-permeabilizing conditions. Insets show vimentin co-localizing with the WGA488 signal in an unpermeabilized GABAergic neuron. **(A,B,G)**: confocal imaging.

So far, two binding partners for C3bot have been identified. The first protein discovered by our group was vimentin which represents an intracellular protein but appears also at the cell surface (Rohrbeck et al., 2014). The second interacting protein residing at the plasma membrane was identified as the ß1-subunit of integrin (Rohrbeck et al., 2017). In the light of these findings, we checked for putative differences in the expression levels of these two proteins in GABAergic vs. glutamatergic neurons that could account for the differential sensitivity of these two neuron subtypes to C3bot. First, we aimed to detect the expression of vimentin and ß1-integrin by using immunofluorescence methods in mixed purified cultures. Both GABAergic and glutamatergic neurons exhibited detectable levels of either protein when co-cultured (Figures 5A,B). Next, we performed qualitative immunofluorescence binding assays using C3bot in mixed GABAergic/glutamatergic cultures. Application of 300 nM of C3bot for 1 h at 4°C revealed a stronger binding of C3bot to GABAergic than glutamatergic neurons (Figure 5C). To allow for a better comparison of the protein levels we performed Western blot assays and stained culture homogenates for vimentin and ß1-integrin in isolated sorted cultures (Figures 5D,E). Whilst ß1-integrin was detected at similar levels in both neuron types vimentin showed a marked reduction of 50% in glutamatergic neurons compared to GABAergic cells. To answer the question of whether the higher vimentin expression in GABAergic neurons was also accompanied by enhanced binding of C3bot to this neuron subtype we performed binding assays in the same cultures used to detect vimentin and ß1-integrin levels. Incubation of GABAergic and glutamatergic neurons with 300 nM at 4°C for 1 h revealed a 30% reduction of C3bot binding to glutamatergic neurons compared to GABAergic cells. To test whether the decreased binding of C3bot could be restituted by the addition of vimentin to glutamatergic neurons we added recombinant vimentin (1 ng/μl) while binding of C3bot was performed (Figure 5F). Quantification revealed that the combinatory treatment of glutamatergic neurons with C3bot and vimentin equalized the amount of bound C3bot in both neuron types, thereby confirming that the observed differences in binding were due to the different vimentin levels.

To demonstrate surface localization of vimentin in GABAergic neurons, living neurons were lectin-treated for 10 min with Alexa488-coupled wheat germ agglutinin (WGA488) to label the plasma membrane. This procedure was then followed by fixation and antibody staining against vimentin in either permeabilized or unpermeabilized cells (Figure 5G). In contrast to permeabilized cells, unpermeabilized GABAergic neurons exhibited a strongly reduced intracellular vimentin signal and a clearly visible co-localization with the WGA488 signal, thus indicating a surface or at least surface-near localization.

In summary, by using a novel transgenic mouse line, the present work demonstrates a differential sensitivity of GABAergic vs. glutamatergic neurons towards the growth-promoting effects of *Clostridium botulinum* C3 protein that is mediated by vimentin.

## Discussion

Vimentin has been demonstrated to function as a key neuronal cell-binding partner for clostridial C3 transferase. In neuronal cell lines such as hippocampal HT22 cells as well as J744A.1 macrophages surface vimentin was the first protein identified to interact with C3bot during the process of cell binding and uptake thereby eliciting its cellular responses (Rohrbeck et al., 2014, 2015). Using a knockout strategy, vimentin was shown to be crucially involved in mediating the growth-promoting effects of C3bot in hippocampal/neocortical neurons (Adolf et al., 2016). In addition to this, site-directed mutagenesis creating mutant C3bot variants with a disturbed RGD integrin-binding domain further identified the ß1-integrin subunit as an additional binding partner for C3bot in neuronal cell lines, macrophages, and primary neuron cultures (Rohrbeck et al., 2017).

Vimentin, besides its role in the formation of the intracellular intermediate filament system, has gained recent attention as a surface-expressed protein, providing a docking structure for invading pathogens in a variety of cell types, amongst them the SARS-associated coronavirus (Mak and Brüggemann, 2016; Yu et al., 2016). Also, this surface expression has been linked to malignant tumor progression (Liu et al., 2020). Various posttranslational modifications such as citrullination or phosphorylation are involved in the regulation of vimentin secretion into the extracellular fluid or in mediating surface presentation in different cell types (for review see Patteson et al., 2020). Also, conformational changes have been identified that elucidate mechanisms of membrane integration as a prerequisite for surface location. In this context, it was demonstrated that vimentin presents as oligomers (4–12 mers) rather than filaments at the surface of glioma cells (Hwang and Ise, 2020). The same group had shown before that the K373-G407 residues of the rod II domain bind *N*-acetylglucosamine and represent a lectin-like cell surface-exposed domain (Ise et al., 2010). On the neuronal surface, the insulin-like growth factor-1 receptor (IGF1R) represents a binding partner for extracellular vimentin that upon activation by vimentin fosters axonal outgrowth in cortical neurons (Shigyo et al., 2015). Current investigations by our group address the question of whether similar mechanisms to the ones found in macrophages, endothelial cells, glioma, or astrocytes that release vimentin by exosomal secretion (Adolf et al., 2019) also exist in neurons. In the developing brain neuronal vimentin is gradually replaced by neurofilaments representing the main intermediate filament system of the mature axon (Li et al., 2013). During the transition from the premature to the differentiated neuron, vimentin was found to be mainly associated with hypophosphorylated neurofilaments to form heteropolymers and to become excluded from extensively phosphorylated neurofilament bundles. In contrast to the vimentin/neurofilament heteropolymers, these bundles stabilize and become resistant to proteolytic cleavage and therefore vimentin is gradually removed from the neurites (Yabe et al., 2003). Generally, the upregulation of (mainly astroglial) vimentin is well documented following CNS lesions in various systems (Pekny and Pekna, 2016). Concomitant with the upregulation, release of glial vimentin to the extracellular space and binding to neuronal membranes may occur (Teshigawara et al., 2013; Shigyo et al., 2015; Shigyo and Tohda, 2016; Adolf et al., 2019). Upregulation of neuronal vimentin, on the other hand, may also take place since it was observed in lesioned sciatic nerve fibers (Perlson et al., 2005) as well as in neurons of the cerebral cortex, cerebellum, and hippocampus affected by Alzheimer’s disease (Levin et al., 2009). In line with this, the injection of kainate into the rat spinal cord resulted in the prolonged neuronal upregulation of vimentin (Nishida et al., 2017). Interestingly, the expression of neuronal vimentin by transfection of differentiated neuroblastoma cells with vimentin resulted in an enhanced axon outgrowth (Dubey et al., 2004). Overall, damage-associated upregulation of vimentin is a proposed response mechanism that recapitulates earlier stages in neuronal development crucial for differentiation and fast process outgrowth. The presentation of an increased amount of extracellular vimentin might be therefore advantageous for the regeneration-promoting properties of C3bot when used as a tool to foster axonal or dendritic re-growth.

Cultured neurons are usually obtained from embryonic or early postnatal days and seem to resemble developing neurons in the *in vivo* situation since they usually express vimentin throughout their culture period (own observations). We here report on a differential expression profile of the intermediate filament protein vimentin but not ß1-integrin between the two major classes of cortical neurons, the projecting excitatory neurons, and the inhibitory interneurons.

Both cell types differ in many anatomical and physiological properties. Moreover, diversity within the lineages of excitatory and inhibitory neurons exists in a way that neocortical excitatory neurons derive from progenitors in the pallium whereas GABAergic interneurons derive from progenitors in the subpallium (Marín and Müller, 2014). Inhibitory GABAergic interneurons *in vivo* comprise only 10–20% of the neuronal population but are characterized by a dense local axonal arbor that coordinates the activity of large populations of local neurons (Booker and Vida, 2018). Despite their relatively low number, they are the most diverse neurons in terms of morphology, connectivity, and physiological properties (Ascoli et al., 2008; Kepecs and Fishell, 2014).

Remarkably, the observed axonotrophic effects of C3bot are more pronounced in GABAergic interneurons even though glutamatergic layer V excitatory neurons, for example, can project to very distant spinal projection areas. The exact reason for the higher vimentin expression in GABAergic neurons remains to be elucidated. Despite the anatomical and physiological differences between inhibitory and excitatory neurons, we could show by the addition of extracellular vimentin that the detected differential sensitivity to C3bot was indeed due to the different vimentin levels. Other integrins such as the β3-subunit have been identified to directly interact with vimentin and to enhance the surface presentation of vimentin at endothelial focal adhesion sites (Bhattacharya et al., 2009). On the other hand, filamentous vimentin underneath the plasma membrane can regulate integrin-ligand interactions by binding to the β3-subunit (Kim et al., 2016). Differential expression profiles of β1-integrins, however, seem not to be responsible for the enhanced vimentin levels in GABAergic neurons or directly account for an increased cell binding of C3bot.

Further work addressing the *in vivo* neuronal growth- and regeneration-promoting properties of C3bot in vimentin knockout mice will add to the understanding of the cellular mechanisms underlying the observed neurotrophic effects.

## Data Availability Statement

The original contributions presented in the study are included in the article, further inquiries can be directed to the corresponding author.

## Ethics Statement

Animal housing as well as all experiments using animal-derived tissue/cells were performed in accordance with institutional (Charité–Universitätsmedizin Berlin Germany), local (LaGeSo, Berlin) and national guidelines (German Animal Welfare Act).

## Author Contributions

AA, PT, and AR performed experiments. IJ, IV, and GA-H contributed to the conception and design of the study. MH and AA designed the study. MH wrote the first draft of the manuscript. All authors contributed to the article and approved the submitted version.

## Conflict of Interest

The authors declare that the research was conducted in the absence of any commercial or financial relationships that could be construed as a potential conflict of interest.
